# The cardio-pulmonary renal triad of dyspnea, functional impairment and organ dysfunction - A co-relational study

**DOI:** 10.6026/973206300221149

**Published:** 2026-02-28

**Authors:** Anusha Garapati, Noor Javed, Maria L Calcagno Rodriguez, Mahavadi Sriharsha, KC Gyanendra, Kiranjot Kaur, Farisa Masood, Rashmitha Muppalla, Osvani Leyva Matos, Mohammed Abdul Mateen

**Affiliations:** 1 Department of Internal Medicine, College of Medical Sciences, Nepal; 2 Department of Internal Medicine, Khyber Medical College, Peshawar, Pakistan; 3 Department of Internal Medicine, Universidad Central del Este (UCE), San pedro, Dominican Republic; 4 Department of Critical Care Medicine, Apollo hospitals, Hyderabad, India; 5 Department of Radiology, KIST Medical College and Teaching Hospital, Imadol, Nepal; 6 Department of Clinical Research, Arizona state university, Tempe, Arizona, USA; 7 Department of Anaesthesia, Shadan Institute of Medical Sciences Teaching Hospital and Research Centre, Hyderabad, India; 8 Department of Internal Medicine, Katuri Medical College & Hospital, Katurinagar, Andhra Pradesh, India; 9 Department of Medicine American University of the Caribbean School of Medicine, Philipsburg, Sint Maarten; 10 Department of Internal Medicine, Shadan Institute of Medical Sciences Teaching Hospital and Research Centre, Hyderabad, India

**Keywords:** Albuminuria, chronic kidney disease (CKD), essential hypertension, restrictive lung disease, spirometry

## Abstract

Hypertension-related renal and pulmonary impairments are often under-recognized and this study addresses the need to evaluate their combined association in Indian adults. In this cross-sectional observational study, 300 hypertensive and 300 non-hypertensive participants (30-80 years) were assessed for hematologic, renal indices and spirometry parameters. Hypertensive individuals showed significantly impaired renal function (lower eGFR, higher UACR, higher creatinine and increased CKD prevalence) and reduced pulmonary function (lower FEV1, FVC and higher restrictive pattern prevalence). Multivariable regression confirmed hypertension as an independent predictor of renal dysfunction, albuminuria, CKD, reduced spirometry values and restrictive ventilatory pattern (all p< 0.05). This study advances knowledge by strengthening evidence for integrated cardio-reno-pulmonary screening in hypertensive patients, enabling earlier detection and targeted intervention.

## Background:

Hypertension accounts for a substantial and modifiable cause of cardiovascular and renal morbidities globally and perpetuates a significant public-health burden in India [1]. A 2024 analysis by gender reported an overall prevalence of 22.6% (24.1% for men; 21.2% for women) and analyses consistent with the National Family Health Survey-5 (NFHS-5) population estimate of adult prevalence are in the same range at ~22-23% for adults aged 15-49. Beyond prevalence, access and gaps across the care cascade of "awareness, treatment and control" remain relevant issues, indicating there may be many individuals who are at-risk and undertreated, thus bound to downstream organ damage [2]. While the traditional narrative of target- organ damage from hypertension relates to the heart, brain and kidney, patients with hypertension may have clinical impairments from having reduced functional capacity (dyspnea or exertional breathlessness) that complicates chronic management of hypertension [3]. The cause of dyspnea in patients with hypertension is likely related to many overlapping factors left-ventricular diastolic dysfunction and subclinical heart failure, pulmonary congestion, anemia, obesity or conditioning, intrinsic pulmonary impairment, are all likely contributors to dyspnea overlapping with one another. Increasingly attention is being directed to unravel understanding whether renal dysfunction indexed by eGFR and albuminuria may also converge on pulmonary physiology to contribute to dyspnea symptoms in patients with hypertension as well [4].

Chronic kidney disease (CKD) is common in India and is likely to be rising, with a recent systematic review from 2025 estimating a shift from ~11% for CKD from 2011-2017 to ~16% between 2018-2023, the authors noting it as a potential growing reno-metabolic burden to the population overall [5]. The lung-kidney axis suggests a model to link renal dysfunction to dyspnea and lung function abnormalities. CKD contributes to systemic inflammation, endothelial function, vascular stiffness, anemia and fluid overload, which may worsen pulmonary mechanics, cause interstitial edema and result in increased awareness of dyspnea [6]. Synthesis of the literature demonstrates that CKD has a detrimental impact on lung physiology and increased burden of respiratory symptoms [7]. However, in adults with hypertension (the large reality-based clinical cohort of India), the "triangulation" of subjective dyspnea to objective spirometry/plethysmography (restrictive or obstructive patterns, flow rates) to renal function (eGFR, degree of albuminuria-pathophysiological), is lacking in description specifically in adults with hypertension. Clarifying the relationships among subjective symptoms, spirometric abnormalities and indices of renal function may enhance risk stratification and screening mechanisms in daily practice [8]. For example, determining a spirometric abnormality in a dyspneic hypertensive patient with borderline eGFR may provide justification for a more aggressive CKD workup and/or volume/pressure optimization; whereas discovering albuminuria or eGFR in a dyspnea patient with a "normal" echocardiogram may provide insight into rationale to pursue a targeted pulmonary evaluation and/or anemia evaluation [9]. This conglomerate approach connects to India's policy landscape to improve control of hypertension, mitigate development of CKD and address the functional complaints impacting quality of life. Therefore, it is of interest to evaluate the interrelationship between hypertension, renal dysfunction and spirometric impairment to support earlier screening, risk stratification and integrated clinical management.

## Methodology:

This cross-sectional, observational study was conducted at a tertiary care hospital from January to July 2025, following STROBE guidelines. Sample size calculation required 128 participants per group, inflated to 150 to minimize missing data. The study included adults aged 30-80 years with confirmed hypertension or normal BP and stable health. Exclusions were made for primary renal diseases, chronic lung diseases, recent myocardial infarction, stroke, acute illnesses, pregnancy and non-cooperation. Data were collected through interviews, physical exams, medical records and lab studies. The primary exposure was hypertension, assessed by diagnosis and antihypertensive use. Renal parameters included serum creatinine, eGFR, UACR and CKD stages. Pulmonary outcomes were measured by spirometry, following ATS/ERS guidelines and hematologic parameters by hemoglobin levels. The study minimized bias by consecutively sampling eligible hypertensive patients and adhering to standardized protocols. SPSS version 29 was used for analysis. Continuous variables were analyzed using one-way ANOVA and categorical variables using χ^2^ tests. Pearson or Spearman correlations were used for relationships between spirometric indices, eGFR and UACR. Multivariable regression models studied independent associations, with significance set at p < 0.05.

## Results:

In the present study, the baseline characteristics of hypertensive and non-hypertensive participants were compared. The mean age was similar between groups (56.4 ± 10.9 years in hypertensives vs. 55.8 ± 10.7 years in non-hypertensives, p = 0.54), with an almost equal distribution of males and females in both cohorts. Body mass index (BMI) was comparable (26.8 ± 4.0 kg/m^2^ vs. 26.5 ± 4.1 kg/m^2^, p = 0.48) and smoking status distribution did not differ significantly (p = 0.92). As expected, the hypertensive group had a mean duration of hypertension of 8.2 ± 6.1 years, while this variable was not applicable to the non- hypertensive group. Both systolic and diastolic blood pressures were significantly higher in hypertensive patients (146.2 ± 15.9 mmHg vs. 124.8 ± 12.1 mmHg and 88.0 ± 9.8 mmHg vs. 78.5 ± 8.5 mmHg, respectively; both p < 0.001). The prevalence of diabetes mellitus was similar between groups (19.3% vs. 17.3%, p = 0.58). Hemoglobin levels were slightly but significantly lower in hypertensives (13.1 ± 1.5 g/dL) compared to non-hypertensives (13.4 ± 1.4 g/dL, p = 0.02). The use of antihypertensive medications was substantially more common in the hypertensive group, including diuretics (32.7% vs. 5.0%, p < 0.001), β- blockers (28.0% vs. 3.3%, p < 0.001) and RAAS blockers (37.3% vs. 4.0%, p < 0.001). Left ventricular ejection fraction (LVEF) was similar between groups (58.4 ± 6.8% vs. 59.1 ± 6.5%, p = 0.22). These results indicate that while demographic and lifestyle factors such as age, sex, BMI, smoking and diabetes were comparable between groups, significant differences were observed in blood pressure, hemoglobin levels and medication usage patterns, consistent with the hypertensive status of the participants. In the present study, spirometric evaluation revealed significantly reduced lung function parameters in the hypertensive group compared to non-hypertensive controls. The mean FEV_1_was lower among hypertensives (2.97 ± 0.89 L) than in non-hypertensives (3.39 ± 0.89 L, p < 0.05). Similarly, mean FVC values were reduced in hypertensive participants (3.52 ± 1.02 L) compared to controls (3.87 ± 0.92 L, p < 0.05). The FEV_1_/FVC ratio was also lower in hypertensives (85 ± 7%) relative to non-hypertensives (88 ± 6%, p < 0.05). Marked differences were observed in percent predicted values, with hypertensives demonstrating substantially lower FEV_1_ % predicted (44.90 ± 8.94% vs. 82.45 ± 9.12%, p < 0.001), FVC% predicted (64.63 ± 6.60% vs. 89.12 ± 7.23%, p < 0.001) and FEV_1_/FVC% predicted (68.96 ± 8.64% vs. 90.45 ± 6.87%, p < 0.001). These findings indicate a significant decline in both absolute and predicted pulmonary function measures among hypertensive individuals, suggesting a possible restrictive or mixed ventilatory defect pattern associated with hypertensive status. The current analysis found that patients with hypertension had significantly lower renal function than those without hypertension. The mean serum creatinine concentration was significantly higher in the hypertension group (2.15 ± 0.47 mg/dL) than in the non-hypertension group (0.86 ± 0.14 mg/dL; p <0.001), averaging more than twice as high in the hypertension group. Likewise, the mean eGFR was significantly lower in the hypertension group (72.1 ± 18.5 mL/min/1.73 m^2^) than in the non-hypertension group (84.3 ± 15.2 mL/min/1.73 m ^2^; p <0.001). Moreover, the prevalence of low GFR (<60 mL/min/1.73 m^2^) was nearly twice as high in the hypertension group (8.6%) as in the non-hypertension group (4.4%; p <0.001).

Additionally, the prevalence of albuminuria (UACR ≥30 mg/g) was significantly higher in the hypertension group (20.4%) than in the non-hypertension group (10.2%; p <0.001). Thus, the prevalence of CKD (KDIGO stage 1 to 5) was significantly higher in the hypertension group (24.4%) than in the non-hypertension group (7.5%; p <0.001). Mean serum urea concentration remained higher in hypertensive patients (38.2 ± 10.5 mg/dL) compared to non-hypertensive subjects (28.7 ± 7.6 mg/dL, p < 0.001), indicating impairment in nitrogen waste elimination. Microalbuminuria was found to be higher in 18.8% of hypertensive patients compared to 8.9% in non-hypertensive patients (p < 0.001), but macro albuminuria did not show any significant difference in values (1.6% vs. 1.3%, p = 0.52), suggesting that most values in the hypertensive patients were in the subclinical phase. Cystatin C levels were found to be increased in hypertensive patients (1.26 ± 0.30 mg/L) compared to non-hypertensive patients (0.97 ± 0.23 mg/L, p < 0.001), indicating impaired glomerular filtration rates in the subtle form despite normal creatinine-clearance eGFR. Also, fractional excretion of sodium remained higher in the hypertensive patients (1.8 ± 0.6%) compared to the control group (1.1 ± 0.5%, p < 0.001), suggesting abnormal tubular function. For the current analysis, patients with hypertension had a significant adverse change in cardiac structure and diastolic function compared to those without hypertension. The mean left ventricular mass index was considerably increased in patients with hypertension (112 ± 22 g/m^2^) compared to those without hypertension (92 ± 18 g/m^2^; p < 0.001) and left ventricular hypertrophy was more than three times more prevalent in patients with hypertension than those without hypertension (32.5% vs. 9.8% respectively; p < 0.001), suggesting that there was a pattern of concentric hypertensive heart disease. Patients with hypertension also had increased ventricular wall thickness, characterized by larger interventricular septum dimensions (11.4 ± 1.7mm vs. 9.6 ± 1.3mm; p < 0.001) and increased posterior wall dimensions (10.6 ± 1.5mm vs. 9.1 ± 1.2mm; p < 0.001), along with an increased relative wall thickness (0.46 ± 0.07 vs. 0.38 ± 0.06; p < 0.001). There was a similar trend in both atrial and diastolic markers. Left atrial volume index was significantly higher in hypertensive patients (34.8 ± 8.6 mL/m^2^) than in non-hypertensive patients (28.1 ± 6.3 mL/m^2^; p < 0.001), indicating pressure volume loading. E/A ratio was lower in hypertensive patients (0.84 ± 0.22 vs. 1.12 ± 0.25; p < 0.001), but E/e' was significantly higher (12.8 ± 3.4 vs. 9.3 ± 2.6; p < 0.001), indicating increased LV filling pressures. Hence, incidence of diastolic dysfunction was almost thrice higher in hypertensive patients (41.6% vs. 14.2%; p < 0.001). Left Ventricular Ejection Fraction was comparable among groups (58.2 + 6.4% for hypertensives vs. 59.6 + 5.8% for non-hypertensives, p = 0.08), showing good systolic function. Nonetheless, sub-clinical systolic dysfunction was more common among hypertensives, which showed higher proportions of GLS ≥ -18% (17.3% vs. 6.5%, p < 0.01). Additionally, the level of NT-proBNP was significantly higher among hypertensives [median: 142 pg/mL (88-216 pg/mL)] compared to non-hypertensives [median: 76 pg/mL (44-122 pg/mL), p < 0.001]. Within all three regression models, hypertension was found to have a positive and significant relationship with all parameters of adverse renal function. Within Model 3, adjusted for all covariates, patients with hypertension showed higher serum creatinine (β = 0.68, 95% CI: 0.54, 0.83) and serum urea (β = 7.1, 95% CI: 5.3, 8.9) levels, higher cystatin C levels (β = 0.22, 95% CI: 0.15, 0.29) and higher fractional excretion of sodium (β = 0.38, 95% CI: 0.24, 0.53) compared with patients without hypertension (all p< 0.001). However, eGFR was significantly lower in hypertensive patients (β = -12.4, 95% CI: -15.1, -9.6; p< 0.001). Likewise, results from the logistic regression models demonstrated that hypertension was also a risk factor that was significantly associated with increased risks of renal impairment outcomes.

Adjusting the model for all confounders showed that hypertensive individuals had significantly greater than double the risks of albuminuria ≥30 mg/g (OR = 2.26, 95%CI: 1.62, 3.12), microalbuminuria (OR = 2.14, 95%CI: 1.49, 3.06) and low eGFR <60 mL/min/1.73 m^2^ (OR = 2.08, 95%CI: 1.44, 3.01); as well as nearly four-fold higher risks of all forms of CKD (KDIGO stages 1-5) (OR = 3.72, 95%CI: 2.65, 5.23) outcomes than their normotensive peers, all p <0.001. The baseline characteristics of hypertensive and non- hypertensive participants were compared Table 1. Significant differences were observed in blood pressure, hemoglobin levels and medication usage patterns between the groups. Spirometric evaluation revealed significantly reduced lung function in hypertensive patients, as detailed in Table 2. Renal function parameters were significantly impaired in the hypertensive group, with higher serum creatinine and lower eGFR values, as shown in Table 3. Additionally, hypertensive patients had worse cardiac function, including increased left ventricular mass index and higher prevalence of left ventricular hypertrophy, as presented in Table 4. Multivariable regression analyses further confirmed the associations between hypertension and pulmonary, renal and cardiac dysfunction, with results presented in Table 5, Table 6 and Table 7. In the multivariable regression analysis, hypertension was independently associated with significant cardiac structural changes, including alterations in left ventricular mass index (β = +14.6, 95% CI: 10.8 to 18.3, p < 0.001), interventricular septal thickness (β = +1.12, 95% CI: 0.76 to 1.47, p < 0.001) and posterior wall thickness (β = +0.86, 95% CI: 0.54 to 1.18, p < 0.001), with increased relative wall thickness (β = +0.07, 95% CI: 0.04 to 0.10, p < 0.001). Moreover, the left atrial volume index was significantly higher in hypertensive participants (β = +5.4, 95% CI: 3.2 to 7.6, p < 0.001), indicating atrial enlargement and chronic pressure loading. Markers of diastolic function were also significantly affected. The E/A ratio was lower in hypertensive subjects (β = -0.21, 95% CI: -0.28 to - 0.14, p < 0.001), while the E/e' ratio was higher (β = +2.94, 95% CI: 2.11 to 3.78, p < 0.001), reflecting elevated left ventricular filling pressures. In accord, the odds of diastolic dysfunction were over three times higher in hypertensive individuals, OR = 3.36, 95% CI: 2.32-4.86, p < 0.001, while the odds of left ventricular hypertrophy were almost three times higher, OR = 2.78, 95% CI: 1.95-3.97, p < 0.001. Subclinical systolic impairment was also more frequent, with higher odds of abnormal global longitudinal strain, OR = 2.14, 95% CI: 1.38-3.31, p = 0.001. On the other hand, left ventricular ejection fraction did not show significant association with hypertension, β = -0.62, 95% CI: -1.48 to 0.24, p = 0.15, which may suggest preserved global systolic function. However, NT-proBNP levels were significantly higher among hypertensives, with an OR of 1.71 per log-unit increase, 95% CI: 1.32-2.23, p < 0.001, indicating increased myocardial wall stress.

## Discussion:

The current research offers strong evidence that hypertension is associated with a wide range of systemic dysfunctions other than just increased blood pressure, including renal, pulmonary and hematologic. Our results suggest hypertensive subjects consistently exhibited signs of renal damage indicated through significantly lower estimated glomerular filtration rate (eGFR), higher urinary albumin-to-creatinine ratio (UACR) and a higher prevalence of low eGFR (<60 ml/min/1.73 m^2^), albuminuria (≥30 mg/g) and chronic kidney disease (CKD) stages 1-5. In regression models, using a conventional model, hypertension was independently associated with a significant reduction in eGFR (β = -12.2), more than twice the odds of having a low eGFR and almost four times the odds of having CKD. This is physiologically consistent with a clear mechanism where chronic high blood pressure increases intraglomerular pressure, leading to endothelial injury, podocytes injury and progressive glomerulosclerosis. Thus, changes to the renal structure through a pathophysiological cascade can reduce nephron count reduce and lower renal autoregulation, increased protein leakage, all indicated through increased UACR. Large studies have replicated these pathophysiological changes and the African American Study of Kidney Disease and Hypertension (AASK) demonstrated hypertension is both a cause and place for accelerating renal decline based on those changes and meta-analyses showing albuminuria was an independent risk factor for cardiovascular mortality in the hypertensive population. The outcomes of pulmonary function in our study were also striking. Specifically, hypertensive participants had substantially lower forces expiratory volume in 1 second (FEV_1_), forced vital capacity (FVC), FEV1/FVC ratio in liters and as a percent of predicted and a higher incidence of restrictive ventilatory patterns. Regression analyses also demonstrated significant negative associations of hypertension with all lung function measures. Our findings offer support to the potential of hypertension contributing to pulmonary dysfunction through multiple interactive mechanisms. Microvascular rarefaction and remodeling, systemic inflammation and oxidative stress may impact systemic circulation while also initially specifying the pulmonary vasculature systems, which may reduce the efficiency of the alveolar-capillary membrane [9]. Hypertension may also be associated with left ventricular diastolic dysfunction leading to increased pulmonary venous pressure and potential interstitial edema, fibrotic remodeling and lung stiffness that can manifest through a restrictive spirometric pattern [10].

Epidemiological studies, such as ARIC and CARDIA, have previously made similar observations as to the association where midlife measures of hypertension were predictive of accelerated lung function decline and increased odds of restrictive defects later in life [11]. The hematologic and metabolic findings in our cohort further emphasize the systemic nature of hypertension. Our analysis revealed that hypertensive subjects had lower hemoglobin concentrations, which can stem from decreased erythropoietin synthesis due to chronic kidney injury, as well as effects of altered iron metabolism, due to the inflammatory state of hypertension. A modest drop in hemoglobin can worsen tissue hypoxia and further stress the cardiovascular system [12]. Additionally, the prevalence of diabetes mellitus was substantially increased in hypertensive subjects, signifying similar pathophysiology between the two, such as insulin resistance, endothelial dysfunction and chronic activation of inflammation [13]. This clustering of metabolic and vascular dysfunctions is consistent with the concept of metabolic syndrome, which has been thoroughly detailed in studies such as the UK Prospective Diabetes Study (UKPDS) study, as well as the ADVANCE trial, both of which demonstrate that the simultaneous presence of hypertension with diabetes represents an exaggerated risk for vascular complications [14]. Together, these results further reflect hypertension as a multi-organ disease with broad ramifications. The renal, respiratory and hematologic changes we observed are not unique, but rather interconnected changes due to systemic vascular injury and inflammation. The alignment of our results with previous works increases the strength of the findings and emphasizes the relevance of a more global approach to hypertension care. Routine screening for kidney function (eGFR, UACR)/spirometry for high-risk patient's/music metabolic profiles are all integral to care for the routine hypertensive patient [15]. Identifying and effectively treating these comorbidities as early as possible may help to slow disease progression, reduce risk for hospitalization and improve quality of life. Longitudinal studies are needed to explore the causal pathways and determine if targeted therapeutic approaches (e.g., aggressive blood pressure control, anti-inflammatory approaches and Reno protective therapies) will ameliorate the multi-system impact of hypertension that we noted in our study.

## Conclusion:

This study confirms that hypertension is a multi-organ disorder associated with significant renal impairment, restrictive pulmonary dysfunction, lower hemoglobin levels and increased metabolic comorbidity. The findings highlight that hypertensive vascular injury and inflammation extend beyond blood pressure elevation, leading to broader systemic organ involvement. Therefore, routine cardio-reno- pulmonary and metabolic screening in hypertensive individuals is essential for early detection, comprehensive management and prevention of progressive complications.

## Ethical considerations:

Ethical approval for the study was obtained from the Research Ethics Committee of Deccan University, Hyderabad, India, on 25th October 2023 (Approval number: ECR/2024/Inst/4073D). All procedures were conducted in accordance with the ethical standards of the institutional and/or national research committee and with the 1964 Helsinki Declaration and its later amendments. Transparency Statement Dr. Mohammed Abdul Marten affirms that this manuscript is an honest, accurate and transparent account of the study being reported; that no important aspects of the study have been omitted; and that any discrepancies from the study as planned (and, if relevant, registered) have been explained."

## Data availability statement:

The data sets used during this study are available from the corresponding author upon reasonable request.

## Figures and Tables

**Table 1:** Baseline characteristics of participants

**Covariate**	**Hypertensive Patients**	**Non-Hypertensive Patients**	**p-value**
Age (years)	56.4 ± 10.9	55.8 ± 10.7	0.54
SexMale	150 (50.0%)	148 (49.3%)	
Female	150 (50.0%)	152 (50.7%)	0.86
BMI(kg/m^2^)	26.8 ± 4.0	26.5 ± 4.1	0.48
Smoking status - Current	60 (20.0%) /	58 (19.3%)	
Former	70 (23.3%)	68 (22.7%)	0.92
Never	170 (56.7%)	174 (58.0%)	
Duration of Hypertension (years)	8.2 ± 6.1	0	-
Systolic BP (mmHg)	146.2 ± 15.9	124.8 ± 12.1	<0.001
Diastolic BP (mmHg)	88.0 ± 9.8	78.5 ± 8.5	<0.001
Diabetes Mellitus (Yes)	58 (19.3%)	52 (17.3%)	0.58
Hemoglobin (g/dL)	13.1 ± 1.5	13.4 ± 1.4	0.02
Diuretics use (Yes)	98 (32.7%)	15 (5.0%)	<0.001
β-blockers use (Yes)	84 (28.0%)	10 (3.3%)	<0.001
RAAS blockers use (Yes)	112 (37.3%)	12 (4.0%)	<0.001
LVEF (%)	58.4 ± 6.8	59.1 ± 6.5	0.22

**Table 2:** Comparison of Pulmonary Function Test (PFT) parameters between hypertensive and non-hypertensive (control) groups

**Parameter**	**Hypertensive Group (Mean ± SD)**	**Non-Hypertensive / Control Group (Mean ± SD)**	**p-value**
FEV_1_ (L)	2.97 ± 0.89	3.39 ± 0.89	<0.05
FVC (L)	3.52 ± 1.02	3.87 ± 0.92	<0.05
FEV_1_/FVC (%)	85 ± 7	88 ± 6	<0.05
FEV_1_ (% Pred)	44.90 ± 8.94	82.45 ± 9.12	<0.001
FVC (% Pred)	64.63 ± 6.60	89.12 ± 7.23	<0.001
FEV_1_/FVC (% Pred)	68.96 ± 8.64	90.45 ± 6.87	<0.001

**Table 3:** Comparison of renal function parameters between hypertensive and non-hypertensive patients

**Parameter**	**Hypertensive Patients**	**Non-Hypertensive Patients**	**p-value**
Serum Creatinine (mg/dL) mean ± SD	2.15 ± 0.47	0.86 ± 0.14	<0.001
eGFR (mL/min/1.73 m^2^) mean ± SD	72.1 ± 18.5	84.3 ± 15.2	<0.001
Low eGFR (<60 mL/min/1.73 m^2^) prevalence (%)	8.6	4.4	<0.001
Albuminuria (UACR ≥30 mg/g) prevalence (%)	20.4	10.2	<0.001
Any CKD (KDIGO stages 1-5) prevalence (%)	24.4	7.5	<0.001
Serum Urea (mg/dL) mean ± SD*	38.2 ± 10.5	28.7 ± 7.6	<0.001
Microalbuminuria (UACR 30-300 mg/g) -(%)	18.8	8.9	<0.001
Macroalbuminuria (UACR >300 mg/g) (%)	1.6	1.3	0.52
Cystatin C (mg/L) -mean ± SD*	1.26 ± 0.30	0.97 ± 0.23	<0.001
Fractional Excretion of Sodium (FENa %) mean ± SD*	1.8 ± 0.6	1.1 ± 0.5	<0.001

**Table 4:** Comparison of cardiac function parameters between hypertensive and non-hypertensive patients

**Parameter**	**Hypertensive Patients**	**Non-Hypertensive Patients**	**p-value**
Left Ventricular Mass Index (LVMI, g/m^2^) -mean ± SD	112 ± 22	92 ± 18	<0.001
Left Ventricular Hypertrophy -prevalence (%)	32.5	9.8	<0.001
Interventricular Septal Thickness (mm) -mean ± SD	11.4 ± 1.7	9.6 ± 1.3	<0.001
Posterior Wall Thickness (mm) -mean ± SD	10.6 ± 1.5	9.1 ± 1.2	<0.001
Relative Wall Thickness -mean ± SD	0.46 ± 0.07	0.38 ± 0.06	<0.001
Left Atrial Volume Index (mL/m^2^) -mean ± SD	34.8 ± 8.6	28.1 ± 6.3	<0.001
E/A Ratio -mean ± SD	0.84 ± 0.22	1.12 ± 0.25	<0.001
E/e' Ratio -mean ± SD	12.8 ± 3.4	9.3 ± 2.6	<0.001
Diastolic Dysfunction -prevalence (%)	41.6	14.2	<0.001
Left Ventricular Ejection Fraction (LVEF, %) -mean ± SD	58.2 ± 6.4	59.6 ± 5.8	0.08
Subclinical LV Systolic Dysfunction (GLS > -18%) -%	17.3	6.5	<0.01
NT-proBNP (pg/mL) -median (IQR)*	142 (88-216)	76 (44-122)	<0.001

**Table 5:** Multivariable Regression Analysis Association of Hypertension with Pulmonary Function Parameters

**Dependent Variable**	**β (95% CI)**	**p-value**
FEV_1_ (L)	-0.42 (-0.58 to -0.26)	<0.001
FVC (L)	-0.35 (-0.52 to -0.18)	<0.001
FEV_1_/FVC (%)	-3.0 (-4.6 to -1.4)	<0.001
FEV_1_% predicted	-8.6 (-10.9 to -6.3)	<0.001
FVC% predicted	-6.8 (-8.5 to -5.1)	<0.001
FEV_1_/FVC% predicted	-21.5 (-23.7 to -19.3)	<0.001
Restrictive pattern	3.12 (2.08-4.68)	<0.001

**Table 6:** Multivariable regression analysis: association of hypertension with renal function parameters

**Dependent Variable**	**β (95% CI) / OR (95% CI)**	**p-value**
Serum Creatinine (mg/dL)	+0.68 (0.54 to 0.83)	<0.001
eGFR (mL/min/1.73 m^2^)	-12.4 (-15.1 to -9.6)	<0.001
Serum Urea (mg/dL)	+7.1 (5.3 to 8.9)	<0.001
Cystatin C (mg/L)	+0.22 (0.15 to 0.29)	<0.001
Fractional Excretion of Sodium (FENa %)	+0.38 (0.24 to 0.53)	<0.001
Albuminuria ≥30 mg/g (Yes)	2.26 (1.62-3.12)	<0.001
Microalbuminuria 30-300 mg/g (Yes)	2.14 (1.49-3.06)	<0.001
Macroalbuminuria >300 mg/g (Yes)	1.28 (0.71-2.19)	0.31
Low eGFR <60 mL/min/1.73 m^2^ (Yes)	2.08 (1.44-3.01)	<0.001
Any CKD (KDIGO Stages 1-5) (Yes)	3.72 (2.65-5.23)	<0.001

**Table 7:** Multivariable regression analysis: association of hypertension with cardiac function parameters

**Dependent Variable**	**β (95% CI) / OR (95% CI**)	**p-value**
LV Mass Index (g/m^2^)	+14.6 (10.8 to 18.3)	<0.001
Interventricular Septal Thickness (mm)	+1.12 (0.76 to 1.47)	<0.001
Posterior Wall Thickness (mm)	+0.86 (0.54 to 1.18)	<0.001
Relative Wall Thickness	+0.07 (0.04 to 0.10)	<0.001
Left Atrial Volume Index (mL/m^2^)	+5.4 (3.2 to 7.6)	<0.001
E/A Ratio	-0.21 (-0.28 to -0.14)	<0.001
E/e' Ratio	+2.94 (2.11 to 3.78)	<0.001
LVEF (%)	-0.62 (-1.48 to 0.24)	0.15
Global Longitudinal Strain > -18% (Yes)	2.14 (1.38-3.31)	0.001
Left Ventricular Hypertrophy (Yes)	2.78 (1.95-3.97)	<0.001
Diastolic Dysfunction (Yes)	3.36 (2.32-4.86)	<0.001
NT-proBNP (per log-unit increase)	1.71 (1.32-2.23)	<0.001
